# Development of a Colloidal Gold Immunochromatographic Test Strip for PPRV Antibody Detection Based on Antigenic Epitope-Derived Recombinant Protein

**DOI:** 10.3390/ani16162545

**Published:** 2026-08-14

**Authors:** Shenyuan Wang, Cong Han, Chuanhao Sun, Dong Zhang, Yongbin Liu

**Affiliations:** 1 Inner Mongolia Autonomous Region Key Laboratory of Biomanufacturing, College of Life Sciences, Inner Mongolia Agricultural University, Hohhot 010018, China; wsy1688521@126.com (S.W.); sunchuanhao1103@163.com (C.S.); 2 College of Grassland Science, Inner Mongolia Agricultural University, Hohhot 010018, China; 15164813299@163.com; 3 China-Mongolia Biomacromolecule Application “Belt and Road” Joint Laboratory, Hohhot 010018, China; 4 Key Laboratory of Mutton Sheep Genetics and Breeding, Ministry of Agriculture and Rural Affairs, Hohhot 010018, China

**Keywords:** peste des petits ruminants virus, Antigenic epitope screening, recombinant protein, Prokaryotic expression, colloidal gold immunochromatographic test strip

## Abstract

Peste des petits ruminants virus (PPRV) causes a highly fatal disease that severely impacts small ruminant production and global food security. This study aimed to develop a rapid, user-friendly colloidal gold immunochromatographic test strip for detecting PPRV-specific antibodies using a double-antigen sandwich format. Bioinformatic analysis using DNASTAR Protean was performed to predict candidate antigenic regions in the PPRV H and N proteins. Three predicted candidate regions from each protein were selected and incorporated into the design of the recombinant fusion antigen PPRV-H_3_N_3_EP. The recombinant antigen was expressed in *E. coli*, purified, and refolded to obtain a final concentration of 8.52 mg/mL. The strip was assembled with colloidal gold-labeled fusion protein as the detection probe and unlabeled protein coated on the test line, plus an independent mouse IgG/goat anti-mouse IgG control system. Performance evaluation showed that the results were readable within 10–15 min. The strip showed satisfactory analytical sensitivity and cross-reactivity performance, consistent qualitative results in within-batch repeatability testing, and preliminary short-term storage stability. In a comparative evaluation using sheep serum samples and a commercial competitive enzyme-linked immunosorbent assay (ELISA) kit, the overall agreement reached 97.9%. Collectively, the constructed PPRV-H_3_N_3_EP antigen enabled a simple, rapid, and reliable strip assay suitable for field detection of PPRV antibodies and post-vaccination monitoring, while also providing a methodological reference for developing antibody tests for other pathogens.

## Simple Summary

Peste des petits ruminants is a deadly viral disease affecting sheep and goats, causing severe losses to farmers and threatening food supplies in many regions. Rapid and simple testing is essential for outbreak control and vaccination monitoring. In this study, we developed a test strip, similar to a pregnancy test, to detect antibodies against this virus in animal blood. We first designed a synthetic protein containing key parts of the virus, produced it in bacteria, and used it to build the test strip. When evaluated using sheep serum samples, the strip provided visually interpretable results within 15 min. No cross-reactivity was observed with the six heterologous positive serum panels tested, and repeated testing using strips from the same batch produced consistent qualitative results. Compared with a commercial competitive enzyme-linked immunosorbent assay, the strip showed an overall agreement of 97.9% in 48 sheep serum samples. This simple, portable device requires no special equipment and can be used on farms to help farmers and veterinarians make quick decisions to protect their herds. It also offers a model for developing similar rapid tests for other animal diseases in the future.

## 1. Introduction

Peste des petits ruminants (PPR) is an acute, highly contagious infectious disease caused by peste des petits ruminants virus (PPRV) [1,2]. Small ruminants such as goats and sheep are the primary susceptible hosts. The disease is mainly transmitted through the respiratory tract via aerosols, while secretions and excretions from infected animals may also contain the virus [3,4]. Clinical manifestations are characterized by sudden high fever (which can persist for several days), anorexia, oral and lingual erosion, severe diarrhea, cough, and bronchopneumonia. In susceptible populations, the disease can spread rapidly through direct contact, with morbidity and mortality reaching 100% and 90%, respectively [5,6]. The disease is now widespread in numerous parts of Africa, the Middle East, South Asia, and East Asia, including China. The World Organisation for Animal Health (WOAH) has designated it as a notifiable animal disease and has proposed a global eradication target by 2030 [7,8]. Given that the clinical symptoms of PPRV are highly similar to those of rinderpest, accurate differential diagnosis is regarded as a critical step to achieve this goal [9,10].

Although PPRV is antigenically classified as a single serotype, molecular phylogenetic analyses have divided PPRV strains into four genetically distinct lineages, designated I–IV, primarily based on partial sequences of the nucleoprotein (N) and fusion (F) genes. These lineages represent genetic diversity rather than distinct serotypes. Therefore, genetic variation among circulating PPRV strains should be considered when selecting viral antigens or epitopes for the development of broadly applicable serological assays [11].

As a member of the genus *Morbillivirus* in the family *Paramyxoviridae*, PPRV contains an approximately 15,948-nt genome composed of non-segmented, negative-sense, single-stranded RNA. The viral particles are enveloped, and the surface spikes are composed of glycoproteins [12,13]. Six structural proteins are encoded by PPRV, among which the nucleocapsid protein (N protein) and the hemagglutinin protein (H protein) are identified as particularly critical mediators of the immune response. The PPRV N protein, the most abundant structural protein of the virus, is responsible for encapsidating the viral RNA to form the nucleocapsid. Multi-target immune regulation is exerted by this protein: interferon regulatory factor 3 and the JAK-STAT signaling pathway are inhibited to suppress type I interferons; NF-κB and the NLRP3 inflammasome are activated to promote the production of interleukin-1β; and autophagy is induced via endoplasmic reticulum stress, phosphoinositide 3-kinase, and key autophagy factors [14,15,16]. These functions make the N protein an important factor in PPRV pathogenesis and immune evasion, as well as a potential target for antiviral intervention [17,18]. The PPRV H protein (approximately 60 kDa) is distributed on the viral surface as a spike glycoprotein, and is identified as the first structural protein that recognizes host receptors during viral invasion [12,19]. By specifically binding to receptors such as signaling lymphocytic activation molecule and nectin-4, the H protein determines the cellular and tissue tropism of the virus [20,21]. At the same time, the H protein activates the membrane fusion activity of the fusion protein by directly interacting with it and inducing its conformational rearrangement. This process facilitates fusion between the viral and host membranes, thereby establishing infection [22,23]. As a surface glycoprotein responsible for receptor binding, the H protein is a major neutralizing antigen and an important mediator of protective humoral immunity [24,25]. Although PPRV is antigenically classified as a single serotype, amino acid variation has been reported among different PPRV strains, including within some antigenic regions of the H protein. In contrast, the N protein is highly immunogenic and generally contains relatively conserved antigenic regions. Therefore, combining selected epitopes derived from both H and N proteins may provide complementary antibody-recognition sites for serological detection.

Currently, diagnostic methods for PPR are mainly divided into four categories: virus isolation, antigen detection, antibody detection, and molecular biological detection [26]. Virus isolation is regarded as a cumbersome procedure that relies on specialized facilities [27]. In terms of detecting antibodies, the virus neutralization test is considered the gold standard; however, it requires exposure to live viruses and involves complex procedures [28,29]. For molecular biology techniques like polymerase chain reaction (PCR) and loop-mediated isothermal amplification (LAMP), PPRV and rinderpest virus can be distinguished using multiplex reverse transcription-PCR. LAMP demonstrates greater sensitivity than RT-PCR in this context. However, these methods rely on professional equipment, and their application in grassroots settings is thus limited [30,31,32]. Colloidal gold immunochromatographic test strips offer several advantages, including simple operation, rapid detection, and no requirement for complex equipment, making them suitable for on-site testing on farms and at local veterinary facilities. Therefore, the development of such rapid, simple, and on-site applicable technologies is regarded as of great significance for the prevention and control of PPR.

According to the different target analytes, colloidal gold immunochromatographic test strips can be divided into several types, including the double-antibody sandwich method, competitive method, and indirect method [33]. Among these, the double-antibody sandwich approach employs two antibodies that bind to distinct epitopes of the same antigen: one is conjugated with colloidal gold, while the other is immobilized on the T line. For the C line, a secondary antibody targeting the gold-labeled antibody is coated to guarantee the accuracy of the assay [34,35]. Based on this principle, Yingjie Bai and colleagues developed a double-antibody sandwich enzyme-linked immunosorbent assay (ELISA) for detecting the spike protein of porcine deltacoronavirus (PDCoV). This method demonstrated high sensitivity and specificity, showed no cross-reactivity with other porcine enteric coronaviruses, and achieved an overall agreement rate of 91.03% when compared to reverse transcription quantitative real-time PCR (RT-qPCR) [36].

In this study, bioinformatic approaches were used to predict candidate antigenic regions in the PPRV H and N proteins. Three predicted candidate regions from each protein were selected and incorporated into the design of the PPRV-H_3_N_3_EP fusion antigen. The resulting recombinant protein was subsequently used to develop a double-antigen sandwich colloidal gold immunochromatographic test strip for PPRV antibody detection. This method offers a straightforward and practical tool for the rapid qualitative detection of PPRV antibodies in serum samples and for assessing the vaccine immune status of flocks. Additionally, it serves as a reference for developing antibody detection methods for other pathogens.

## 2. Materials and Methods

### 2.1. Serum Samples

A total of 48 serum samples were collected from 48 seven-month-old sheep at Inner Mongolia Shengle Biotechnology Co., Ltd. (Hohhot, China), with each sample obtained from a different animal. All sheep had been immunized with a live attenuated peste des petits ruminants vaccine (Clone9 strain; Tecon Bio-pharmaceutical Co., Ltd., Urumqi, China). Blood samples were collected 28 days after vaccination. Briefly, 10 mL of blood was collected from the jugular vein of each sheep and allowed to clot at room temperature for 30 min. The samples were then centrifuged at 1300× *g* rpm for 15 min at 4 °C using a refrigerated centrifuge (Beckman Coulter, Brea, CA, USA). The upper serum layer was carefully collected, aliquoted, and stored at −20 °C until use.

For the cross-reactivity evaluation, positive sera against foot-and-mouth disease (FMD), sheep pox (SP), brucellosis (Bru), pasteurellosis (Past), mycoplasmal pneumonia (MP), and streptococcosis (Strep) were also obtained from Inner Mongolia Shengle Biotechnology Co., Ltd. Sera from two-week-old unvaccinated lambs maintained at the Inner Mongolia Key Laboratory of Biomanufacturing (Hohhot, China) were used as PPRV-negative control sera and stored at −20 °C until use. Because PPRV vaccination is included in the national compulsory animal immunization program in China, including its implementation in Inner Mongolia Autonomous Region, most sheep in routine production systems in the sampling area had a history of PPRV vaccination. Consequently, only a limited number of well-characterized serum samples from unvaccinated, PPRV-antibody-negative animals were available for the present study.

### 2.2. Construction of the pET-28a-PPRV-H_3_N_3_EP Recombinant Plasmid

#### 2.2.1. Viral Protein Sequences and Bioinformatics Analysis

The amino acid sequences of the H protein (Protein ID: AEH25644.1) and N protein (Protein ID: AEH25639.1) of the lineage IV PPRV isolate China/Tib/07 (GenBank accession no. JF939201.1) were obtained from GenBank. Candidate antigenic regions were predicted using Protean 3D (Lasergene version 17.6, DNASTAR, Madison, WI, USA). The prediction profiles included antigenicity assessed using the Jameson-Wolf and Welling methods, flexibility assessed using the Karplus-Schulz method, hydropathy assessed using the Kyte-Doolittle method, and protein stability. Candidate regions showing relatively favorable antigenicity profiles were further evaluated together with their hydropathy and stability characteristics. Based on these prediction results, three candidate regions were selected from each protein. The selected regions were located at residues 195–214, 398–425, and 496–515 of the H protein and at residues 53–73, 103–122, and 239–258 of the N protein, and were designated H2, H16, H1, N14, N1, and N2, respectively. These predicted candidate regions were subsequently incorporated into the design of the PPRV-H3N3EP fusion antigen. Detailed information on the selected regions is provided in Supplementary Table S1, and the DNASTAR Protean prediction profiles are shown in Supplementary Figure S1. The physicochemical properties of PPRV-H3N3EP were subsequently analyzed using the ProtParam tool on the ExPASy platform (ExPASy, SIB Swiss Institute of Bioinformatics; https://web.expasy.org/protparam/, accessed on 12 August 2026).

#### 2.2.2. Construction of the Recombinant Plasmid

After codon optimization, the PPRV-H3N3EP gene was synthesized by Sangon Biotech (Shanghai) Co., Ltd. (Shanghai, China) and inserted into the XhoI and NcoI restriction sites of the pET-28a(+) vector (TaKaRa, Beijing, China).

#### 2.2.3. Transformation and Amplification of the Recombinant Plasmid

The recombinant plasmid was transformed into *E. coli* DH5α competent cells using the heat-shock method. Briefly, the competent cells were transferred directly from ice to a 42 °C water bath and heat-shocked for 90 s, followed by immediate cooling on ice for 2 min. The cells were handled gently without agitation throughout the heat-shock procedure. These cells were subsequently plated onto Luria–Bertani (LB) agar medium supplemented with kanamycin sulfate at a concentration of 0.05 g/L (obtained from Solarbio, Beijing, China), and incubated overnight at 37 °C. Individual colonies were selected and used to inoculate LB liquid medium supplemented with kanamycin sulfate. The cultures were then incubated with shaking at 37 °C and 180 rpm for 12 h.

#### 2.2.4. Extraction and Identification of the Recombinant Plasmid

The recombinant plasmid was extracted using a plasmid miniprep kit (Tiangen, Beijing, China) according to the manufacturer’s instructions, and its concentration was adjusted to 800 ng/μL. The plasmid was then identified via colony PCR and double restriction enzyme digestion. The PCR primers (Forward: 5′-ATGGGCAGCAGCCATCATCAT-3′; Reverse: 5′-TCAGTGGTGGTGGTGGTGGT-3′) were synthesized by Sangon Biotech (Shanghai) Co., Ltd., and the amplified product had a size of 630 bp. Ex Taq polymerase (TaKaRa, Beijing, China) was used for the PCR reaction, with an annealing temperature of 61 °C. For double enzyme digestion, XhoI and NcoI (TaKaRa, Beijing, China) were used, and the reaction was performed at 37 °C for 1.5 h. After verification via agarose gel electrophoresis, the correctly identified recombinant plasmid was commissioned to Sangon Biotech (Shanghai) Co., Ltd. for sequencing identification.

### 2.3. Prokaryotic Expression and Identification of the PPRV-H_3_N_3_EP Protein

#### 2.3.1. Transformation of the Recombinant Plasmid into Expression Host Bacteria

The correctly identified recombinant plasmid was transformed into *E. coli* BL21(DE3) competent cells (TransGen, Beijing, China) using the heat-shock method. Briefly, the competent cells were transferred directly from ice to a 42 °C water bath and heat-shocked for 90 s, followed by immediate cooling on ice for 2 min. The cells were handled gently without agitation throughout the heat-shock procedure. Individual colonies were subsequently selected, mixed with glycerol to a final concentration of 30%, and stored at −80 °C as glycerol stocks.

#### 2.3.2. Expression of the PPRV-H_3_N_3_EP Protein

The glycerol stocks were inoculated into LB liquid medium supplemented with kanamycin at a ratio of 1:100 and cultured at 37 °C and 220 rpm in a temperature-controlled orbital shaker (ZWY-110X30, Shanghai Zhicheng Analytical Instrument Manufacturing Co., Ltd., Shanghai, China) until the optical density at 600 nm reached 0.6–0.8. Isopropyl β-D-1-thiogalactopyranoside (IPTG) was then added to a final concentration of 0.1 mM, and protein expression was induced at 10 °C and 170 rpm for 8 h using the same shaker. After induction, the bacterial cultures were harvested for subsequent isolation and purification of the inclusion bodies.

#### 2.3.3. Purification and Refolding of Inclusion Bodies

The inclusion bodies were solubilized in binding/wash buffer containing 4 M urea and purified using Ni-NTA 6FF affinity chromatography (Sangon Biotech, Shanghai, China). The recombinant protein was eluted using an imidazole gradient of 20–400 mM, and fractions containing the target protein were collected. The purified protein was subsequently refolded by gradual reduction in the urea concentration from 4 M to 0 M in the presence of reduced and oxidized glutathione. The refolded protein was concentrated by ultrafiltration and quantified using the bicinchoninic acid assay (Solarbio, Beijing, China).

#### 2.3.4. Western Blot Analysis

The purified and concentrated PPRV-H3N3EP protein was diluted to an appropriate concentration, mixed with 5× loading buffer at a ratio of 4:1, and denatured at 95 °C. The samples were then subjected to sodium dodecyl sulfate–polyacrylamide gel electrophoresis (SDS-PAGE) at 80 V through the stacking gel and 120 V through the separating gel. Following electrophoresis, the gel was trimmed according to the protein molecular weight marker (Thermo Fisher Scientific, Waltham, MA, USA), and a polyvinylidene fluoride membrane (Biosharp, Hefei, China) was cut to the corresponding size. The membrane was initially treated with methanol for 1 min, after which a semi-dry transfer was performed at 150 mA for 10 min using a transfer device (Bio-Rad Laboratories, Hercules, CA, USA). The membrane was incubated in a blocking solution containing 5% skim milk for 1.5 h at room temperature. Following this, it was rinsed four times using Tris-buffered saline with Tween (TBST), with each wash lasting 10 min. Serum positive for anti-PPRV antibodies, derived from sheep immunized with the PPRV vaccine, was diluted 1:8000 and used as the primary antibody. The membrane was initially incubated with the serum for 1.5 h at room temperature, after which it was kept overnight at 4 °C. After five washes using TBST, the membrane was then treated with horseradish peroxidase-conjugated rabbit anti-sheep immunoglobulin G (IgG) (ABclonal Biotechnology Co., Ltd., Wuhan, China) at a 1:6000 dilution for 2 h at ambient temperature. The membrane was subsequently rinsed five additional times with TBST. An enhanced chemiluminescence detection kit (Thermo Fisher Scientific, Waltham, MA, USA) was used for signal development, and images were captured using a chemiluminescence imaging system (GE Healthcare Bio-Sciences AB, Uppsala, Sweden).

### 2.4. Preparation of the Colloidal Gold Immunochromatographic Test Strip

#### 2.4.1. Synthesis of Colloidal Gold and Optimization of Labeling Conditions

Colloidal gold was synthesized using the trisodium citrate reduction method. Briefly, 100 mL of ultrapure water was brought to a boil, followed by the addition of 1 mL of 1% chloroauric acid (Tianjin Maisike Chemical Co., Ltd., Tianjin, China). After 2.5 min, 2 mL of 1% trisodium citrate (Fuchen (Tianjin) Chemical Reagent Co., Ltd., Tianjin, China) was rapidly added under continuous stirring. The solution gradually changed from light yellow to grayish purple and finally to transparent wine red. After cooling, the final volume was adjusted to 100 mL. The colloidal gold particles were characterized by ultraviolet-visible spectrophotometry (Techcomp, Beijing, China) and transmission electron microscopy (Wuhan Maisipu Biotech, Wuhan, China).

In this experiment, the checkerboard titration technique was employed to identify the most suitable pH level and protein concentration for labeling the PPRV-H_3_N_3_EP protein with colloidal gold. The protein was diluted to 1 mg/mL with phosphate-buffered saline, and each component was added into the wells of a microtiter plate according to Table 1 Different pH conditions for the colloidal gold solution were established by adding 0.1 M K_2_CO_3_. For each condition, 125 μL of the treated solution was transferred into individual wells, gently mixed, and incubated for 15 min at room temperature. An aliquot of 125 μL of 10% NaCl was then introduced, and the resulting mixture was left undisturbed for 15 min. Color change from wine red to grayish purple was regarded as aggregation, while maintaining red was regarded as stable. Combined with the optical density at 520 nm (OD_520_) readings, the minimum protein dosage required to maintain a stable red color at each pH was determined. The maximum OD_520_ value of the optimal well at each pH was compared, and the optimal labeling pH and the corresponding protein dosage were screened. The theoretical optimal labeling dosage for 1 mL of colloidal gold was calculated as the protein dosage of this well multiplied by 8, and the actual protein dosage used for labeling was 120% of the theoretical optimal dosage.

The optimal labeling pH and protein dosage for mouse IgG were determined using the same procedure described above.

#### 2.4.2. Colloidal Gold Labeling of Proteins

1 mL of colloidal gold solution at the optimal pH was taken, and the target protein was added at the optimal labeling dosage, followed by vortex mixing and incubation at room temperature for 90 min. Then 10% bovine serum albumin (BSA, Guangzhou Saiguo Biotechnology Co., Ltd., Guangzhou, China) was incorporated to achieve a final concentration of 1% for blocking purposes, and the mixture was subsequently incubated at room temperature for 45 min. For stabilization, polyethylene glycol 20,000 (PEG 20000; Biosharp, Hefei, China) was added from a 5% solution to a final concentration of 0.1%, followed by incubation at room temperature for 45 min. Following centrifugation at 12,000× *g* rpm for 15 min at 4 °C, the supernatant was carefully removed. The pellet was washed with gold-labeling resuspension buffer (50 mM Tris-HCl, 1% BSA, pH 8.0) and subjected to centrifugation once again using the same parameters. The supernatant was discarded, and two additional washes were carried out. The pellet was resuspended in 100 μL of gold-labeling resuspension buffer by repeated pipetting until no visible aggregates remained. The resulting 10× concentrated gold-labeled protein preparation was stored at 4 °C in the dark until use. Mouse IgG (Solarbio, Beijing, China) was labeled using the same method.

#### 2.4.3. Preparation and Assembly of the Colloidal Gold Immunochromatographic Test Strip

The lateral flow strip was prepared by sequentially arranging a glass fiber sample pad (SB-08, Shandong Kelaide Biotechnology Co., Ltd., Shandong, China), a conjugate pad (G-4), a nitrocellulose (NC) membrane (CN140, Sartorius, Göttingen, Germany), and an absorbent pad (H-1) onto a plastic backing plate. The backing plate was prepared by placing the NC membrane at its center. The PPRV-H_3_N_3_EP protein (T line, 3 mg/mL) and goat anti-mouse IgG (Solarbio, Beijing, China) (C line, 2 mg/mL) were coated onto the NC membrane using a three-dimensional gold-spraying and membrane-spotting instrument (XYZ3000 dispensing platform, Biodot, Irvine, CA, USA). The membrane was first equilibrated at room temperature for 30 min, followed by drying at 37 °C for 3.5 h. It was then sealed with desiccant in a self-sealing bag and preserved at 4 °C for subsequent use. The gold-conjugated PPRV-H_3_N_3_EP protein was combined with gold-conjugated mouse IgG at an optimized ratio and subsequently immobilized on the conjugate pad. After sequential assembly of the sample pad, conjugate pad, NC membrane, and absorbent pad, the prepared strips were sealed and kept in a dry environment at 4 °C.

In the double-antigen sandwich detection system, the serum specimen was first dispensed onto the sample pad. In samples containing antibodies against PPRV, the antibodies specifically bound to the gold-conjugated antigen, resulting in the formation of antigen–antibody complexes. During lateral flow migration, the antigen–antibody complex was specifically captured by the immobilized unlabeled antigen at the test line, thereby producing a visible red band. Meanwhile, the gold-conjugated mouse IgG migrated toward the C line and was captured by the immobilized goat anti-mouse IgG, producing a visible control band. The test was considered valid when the C line produced a visible colored band. The presence of bands at both the T and C lines represented a positive result, while a single band appearing only at the C line was interpreted as negative.

### 2.5. Colloidal Gold Test Strip Assay Procedure

#### 2.5.1. Analytical Sensitivity

The PPRV antibody-positive serum was serially diluted (1:2 to 1:128) and detected using the test strip, with the detection repeated 3 times.

#### 2.5.2. Cross-Reactivity

Positive sera for PPRV, FMD, SP, Bru, Past, MP, and Strep were used for detection, with the detection repeated 3 times.

#### 2.5.3. Within-Batch Repeatability

Ten serum samples, including five positive and five negative samples according to the commercial ELISA, were tested using strips from the same production batch (batch no. 20250728). Each serum sample was tested in triplicate under the same experimental conditions, and the qualitative positive/negative results among the three replicates were compared.

#### 2.5.4. Preliminary Short-Term Storage Stability

Preliminary short-term storage stability: The test strips were stored at 4 °C or room temperature (approximately 25 °C) and evaluated weekly using positive and negative sera to monitor changes in their qualitative detection performance. This experiment was conducted as a preliminary short-term storage assessment rather than a formal shelf-life study.

### 2.6. Comparison with Commercial ELISA Kit

To evaluate agreement between the developed test strip and a commercial serological assay, the 48 sheep serum samples were tested in parallel using the developed strip and a commercial competitive ELISA kit for PPRV antibody detection (Competitive ELISA Kit for Detecting Antibody of Peste Des Petits Ruminants, Lanzhou Shouyan Biotechnology Co., Ltd., Lanzhou, China). The ELISA was performed and interpreted according to the manufacturer’s instructions. The commercial ELISA was used as a comparator assay rather than an absolute reference standard.

### 2.7. Statistical Analysis

The commercial competitive ELISA was used as the comparator assay for evaluating agreement with the developed test strip. A 2 × 2 contingency table was constructed based on the results obtained using the two methods. Positive percent agreement (PPA), negative percent agreement (NPA), and overall percent agreement (OPA) were calculated as follows: PPA = a/(a + c) × 100%, NPA = d/(b + d) × 100%, and OPA = (a + d)/N × 100%, where a represents samples positive by both methods, b represents strip-positive/ELISA-negative samples, c represents strip-negative/ELISA-positive samples, and d represents samples negative by both methods. Positive predictive value (PPV) and negative predictive value (NPV), relative to the commercial ELISA comparator, were calculated as a/(a + b) × 100% and d/(c + d) × 100%, respectively. Exact binomial 95% confidence intervals (CIs) were calculated for these estimates. Agreement between the two methods was further assessed using Cohen’s kappa coefficient. Statistical analyses were performed using IBM SPSS Statistics version 26.0 (IBM Corp., Armonk, NY, USA). A two-sided *p* value < 0.05 was considered statistically significant.

## 3. Results

### 3.1. Design of the Recombinant Protein PPRV-H_3_N_3_EP and Plasmid Construction

#### 3.1.1. Bioinformatic Prediction and Physicochemical Characteristics of PPRV-H3N3EP

Bioinformatic analysis of the H and N proteins using DNASTAR Protean identified multiple regions with relatively favorable predicted antigenicity profiles. Based on the antigenicity profiles together with hydropathy and stability characteristics, three candidate regions were selected from each protein. The selected regions corresponded to residues 195–214 (H2), 398–425 (H16), and 496–515 (H1) of the H protein and residues 53–73 (N14), 103–122 (N1), and 239–258 (N2) of the N protein. The DNASTAR Protean prediction profiles are shown in Supplementary Figure S1, and the positions and amino acid sequences of the six selected candidate regions are summarized in Supplementary Table S1. The physicochemical features of the fusion gene-encoded PPRV-H3N3EP protein were analyzed using ProtParam on the ExPASy server. The protein was predicted to contain 209 amino acid residues, with an estimated molecular mass of 21.49 kDa, a theoretical pI of 6.06, and a molecular formula of C_914_H_1467_N_283_O_301_S_8_. With an aliphatic index of 84.35 and an instability index of 28.98 (<40), the protein was predicted to be stable. The estimated half-life was 30 h in mammalian reticulocytes, >20 h in yeast, and >10 h in *Escherichia coli*.

Based on the above design, the recombinant plasmid pET-28a-PPRV-H_3_N_3_EP containing the fusion gene was synthesized and constructed.

#### 3.1.2. Identification of the pET-28a-PPRV-H_3_N_3_EP Recombinant Plasmid

**Figure 1:**
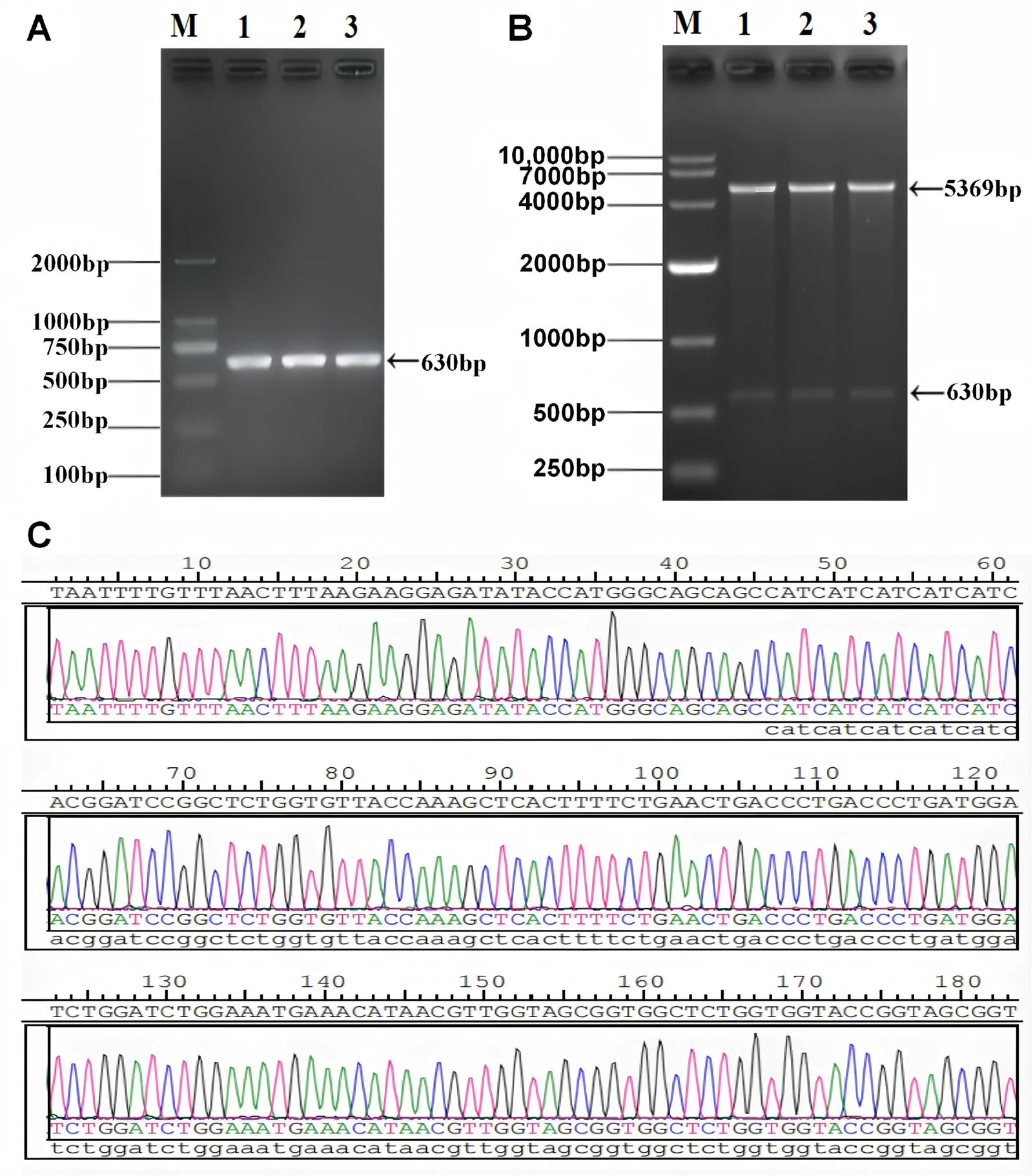
Identification of the pET-28a-PPRV-H_3_N_3_EP recombinant plasmid. (**A**) Bacterial suspension PCR verification. M: DNA marker; lanes 1–3: PCR products from bacterial suspensions. (**B**) Double restriction enzyme digestion of the recombinant plasmid. M: DNA marker; lanes 1–3: products digested with XhoI and NcoI. (**C**) Partial sequencing chromatogram of the recombinant plasmid.

The results of colony PCR of the bacteria containing the pET-28a-PPRV-H_3_N_3_EP recombinant plasmid showed that the target fragment consistent with the expected size was amplified (630 bp, Figure 1A). The results of double restriction enzyme digestion with XhoI and NcoI showed that two specific bands were obtained, which corresponded to the 630 bp target gene fragment and the 5369 bp linearized vector, respectively (Figure 1B). The successful construction of the recombinant plasmid was further confirmed by sequencing (Figure 1C).

### 3.2. Recombinant Expression, Purification, and Western Blot Identification of the PPRV-H3N3EP Protein

**Figure 2:**
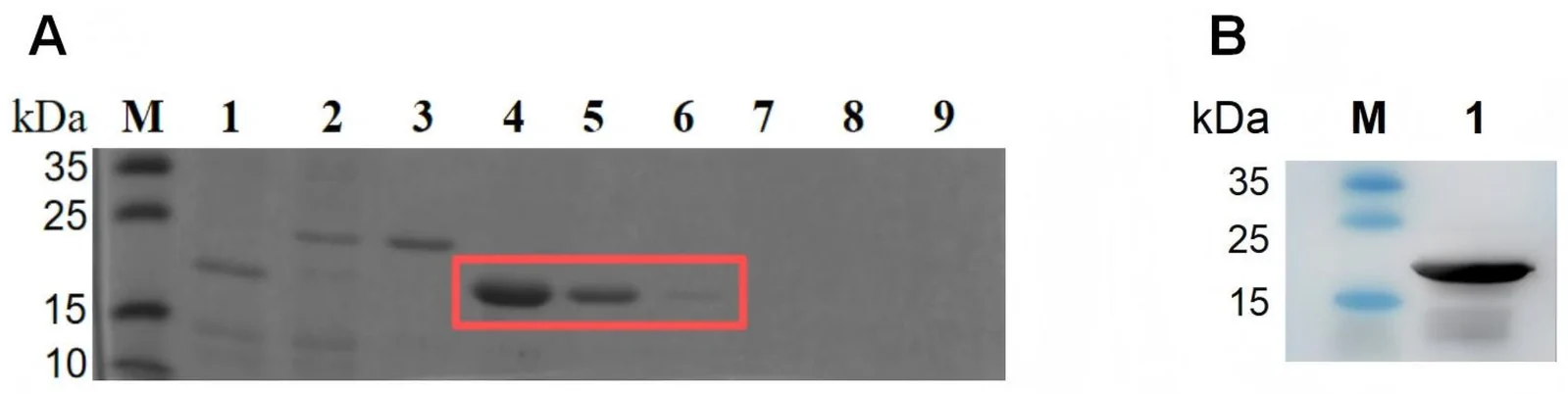
Purification and Western blot identification of the recombinant PPRV-H_3_N_3_EP protein. (**A**) SDS-PAGE analysis of the purified recombinant protein. M, protein molecular weight marker; lane 1, binding/wash buffer flow-through; lanes 2–9, elution fractions collected using 20, 50, 100, 150, 200, 250, 300, and 400 mM imidazole, respectively. The target band corresponding to the recombinant PPRV-H_3_N_3_EP protein appeared at approximately 21.5 kDa. (**B**) Western blot identification of the recombinant protein. M, protein molecular weight marker; lane 1, purified PPRV-H_3_N_3_EP protein probed with PPRV-positive sheep serum.

After transformation into *E. coli* BL21(DE3), the recombinant PPRV-H3N3EP protein was expressed under the induction conditions described in the Materials and Methods. Following purification by Ni-NTA affinity chromatography under denaturing conditions and subsequent refolding, SDS-PAGE analysis showed that the target protein was mainly present in the fractions eluted with 100, 150, and 200 mM imidazole. A prominent band was observed at approximately 21.5 kDa, consistent with the predicted molecular weight of PPRV-H3N3EP (Figure 2A, the area marked by the red box). Western blot analysis using PPRV-positive sheep serum revealed a single immunoreactive band at approximately 21.5 kDa, confirming the immunoreactivity of the recombinant protein (Figure 2B).

### 3.3. Development of the Colloidal Gold Immunochromatographic Test Strip for PPRV Antibody Detection

#### 3.3.1. Characterization of Colloidal Gold and Optimization of Protein Labeling Conditions

**Figure 3:**
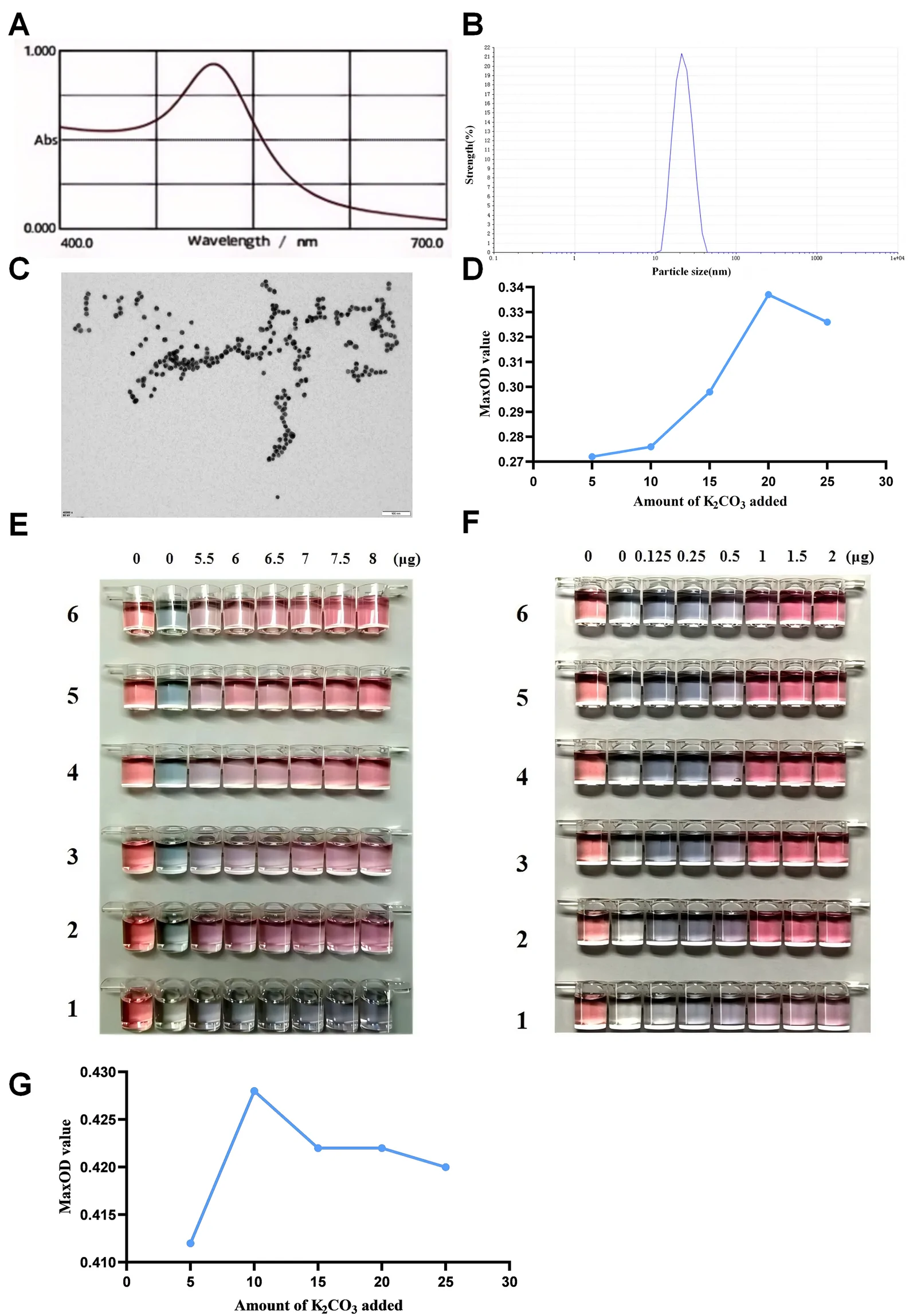
Characterization of colloidal gold and optimization of labeling conditions. (**A**) Visible absorption spectrum of colloidal gold. (**B**) Intensity-based size distribution of colloidal gold. (**C**) Transmission electron microscopy image of colloidal gold. (**D**) Determination of optimal labeling conditions for PPRV-H_3_N_3_EP protein. (**E**) Determination of optimal labeling amount of PPRV-H_3_N_3_EP protein under different K_2_CO_3_ concentrations, where the amounts of PPRV-H_3_N_3_EP protein added per column are 0, 0, 5.5, 6, 6.5, 7, 7.5, 8 μg (from left to right), and rows 1–6 correspond to 0.1 M K_2_CO_3_ volumes of 0, 5, 10, 15, 20, 25 μL (from top to bottom). (**F**) Determination of optimal labeling amount of mouse IgG under different K_2_CO_3_ concentrations, where the amounts of mouse IgG added per column are 0, 0, 0.125, 0.25, 0.5, 1, 1.5, 2 μg (from left to right), and rows 1–6 correspond to 0.1 M K_2_CO_3_ volumes of 0, 5, 10, 15, 20, 25 μL (from top to bottom). (**G**) Determination of optimal labeling conditions for mouse IgG.

Ultraviolet-visible spectroscopy showed a single narrow absorption peak at 520 nm (Figure 3A). Particle-size analysis indicated a mean particle size of 22.35 nm and a polydispersity index of 0.162 (Figure 3B). Transmission electron microscopy revealed predominantly round or oval particles with an average diameter of approximately 22 nm, good dispersion, and no obvious aggregation (Figure 3C).

On this basis, to optimize the labeling dosage of the PPRV-H_3_N_3_EP protein, checkerboard titration was performed under 6 pH conditions (with 0, 5, 10, 15, 20, and 25 μL of 0.1 M K_2_CO_3_ added, respectively), and the optimal conditions were determined based on color stability and OD520 values. The results showed that the condition with 0 μL of K_2_CO_3_ was not suitable for labeling. When 5–20 μL of K_2_CO_3_ was added, the optimal labeling dosage was 60 μg/mL colloidal gold, while it was 56 μg/mL when 25 μL of K_2_CO_3_ was added (Figure 3E). By comparing the maximum OD value of the optimal well at each pH, the optimal labeling conditions were determined. The highest OD value was obtained with the addition of 20 μL K_2_CO_3_ and 60 μg protein. Therefore, the optimal conjugation amount of PPRV-H_3_N_3_EP protein was determined to be 72 μg/mL of colloidal gold (60 μg × 120%) (Figure 3D).

The same pH conditions were used to screen the optimal labeling dosage of mouse IgG. The results showed that except for the 0 μL group, which was not suitable for labeling, the optimal labeling dosage was 8 μg/mL colloidal gold when 5–20 μL of K_2_CO_3_ was added, while it was 12 μg/mL when 25 μL of K_2_CO_3_ was added (Figure 3F). The comparison of the maximum OD values showed that the maximum OD value was obtained when the K_2_CO_3_ addition amount was 10 μL and the mouse IgG addition amount was 8 μg. Therefore, the actual optimal labeling dosage of mouse IgG was determined as 9.6 μg/mL colloidal gold (8 μg × 120%) (Figure 3G).

#### 3.3.2. Performance Evaluation of the Colloidal Gold Test Strip

**Figure 4:**
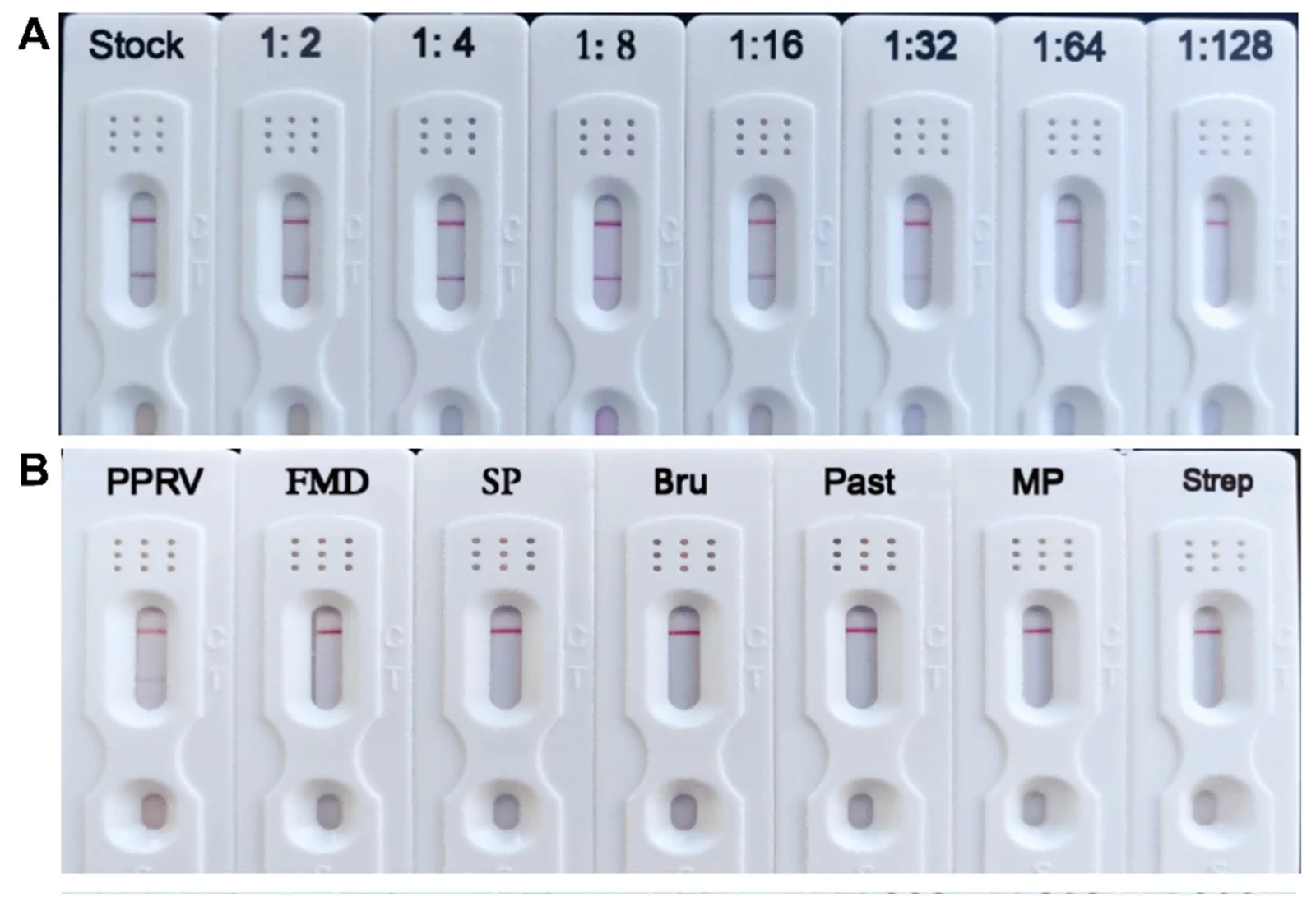
Comprehensive evaluation of the colloidal gold test strip performance. (**A**) Analytical sensitivity of the colloidal gold immunochromatographic strip for detecting PPRV-positive serum at two-fold serial dilutions from 1:2 to 1:128. (**B**) Cross-reactivity evaluation of the colloidal gold immunochromatographic strip.

To determine the sensitivity of the test strip, PPRV-positive serum was subjected to twofold serial dilution from 1:2 to 1:128 and then tested. The results showed that when the undiluted PPRV-positive serum was tested, clear red bands were observed at both the T and C lines. With the increase in the dilution factor, the band intensity of the T line gradually decreased; a faint band was observed when the serum was diluted to 1:64, while the T line was no longer visible at a dilution of 1:128 (Figure 4A). In the cross-reactivity evaluation, positive sera for PPRV, FMD, SP, Bru, Past, MP, and Strep were detected using the prepared test strip, respectively. The results demonstrated that a visible band appeared at the T line only in the PPRV-positive detection, whereas no color signal was detected at the T line in the other tests (Figure 4B). For the within-batch repeatability evaluation, 10 serum samples, including five ELISA-positive and five ELISA-negative samples, were each tested in triplicate using test strips from the same production batch (batch no. 20250728). All 30 replicate tests produced qualitative results consistent with the corresponding expected positive or negative classification, resulting in 100% within-batch qualitative concordance (30/30) under the conditions evaluated (Table 2). In the preliminary short-term storage stability evaluation, the test strips were stored at 4 °C or room temperature (approximately 25 °C) and tested weekly using positive and negative sera. After 5 weeks of storage at room temperature, the T- and C-line bands became indistinct or were no longer clearly visible. In contrast, the bands remained clearly distinguishable after 8 weeks of storage at 4 °C. These findings indicate better short-term preservation of the test strips at 4 °C under the conditions evaluated; however, the present experiment was not designed to establish the shelf life of the product. A total of 48 sheep serum samples were evaluated using both the developed test strip and the commercial competitive ELISA (Table 3). Among these samples, 45 were positive by both methods, two were negative by both methods, and one was positive by the commercial ELISA but negative by the developed test strip. No sample was positive by the developed strip but negative by the commercial ELISA. Accordingly, relative to the commercial ELISA comparator, the developed test strip showed a positive percent agreement (PPA) of 97.8% (45/46; 95% CI: 88.5–99.9%), a negative percent agreement (NPA) of 100% (2/2; 95% CI: 15.8–100%), and an overall percent agreement (OPA) of 97.9% (47/48; 95% CI: 88.9–99.9%). Cohen’s kappa coefficient was 0.789 (*p* < 0.0001). However, because only two ELISA-negative samples were included, the NPA should be interpreted with caution and should not be considered a definitive estimate of diagnostic specificity. In addition, because the original sample-level mapping between the ELISA results and the individual serum identification numbers could not be reliably recovered, the identification number of the single discordant sample could not be determined.

## 4. Discussion

As a WOAH-listed notifiable animal disease, PPR poses a major risk to the global sheep industry. Therefore, rapid, accurate, and convenient serological detection methods are of great significance for the early warning, epidemic surveillance, and evaluation of vaccine immune efficacy of PPR [37].

In this study, the PPRV-H3N3EP fusion antigen was designed by incorporating six predicted candidate antigenic regions derived from the PPRV H and N proteins. The H protein is a major neutralizing antigen of PPRV, whereas the N protein is highly immunogenic [38,39]. Therefore, the rationale for incorporating sequences derived from both proteins was to provide multiple potential antibody-recognition regions within a single recombinant antigen. The candidate regions were selected based on DNASTAR Protean prediction profiles, including antigenicity, hydropathy, flexibility, and stability-related characteristics. Flexible glycine-serine-rich linkers were incorporated into the fusion construct to reduce potential steric interference between adjacent regions [40]. Western blot analysis demonstrated that the recombinant PPRV-H3N3EP protein reacted with PPRV-positive sheep serum, supporting the overall immunoreactivity of the fusion protein. However, this result does not demonstrate the individual antigenicity or relative contribution of each predicted candidate region. Therefore, the present findings support the immunoreactivity of PPRV-H3N3EP as an integrated fusion antigen rather than experimentally validating each predicted region individually.

Based on the above design, the PPRV-H3N3EP-based colloidal gold immunochromatographic test strip was developed and its performance was evaluated. Analytical sensitivity testing showed that PPRV-positive serum remained detectable at a dilution of 1:64 under the conditions used in the present study. Various serological assay formats have previously been developed for PPRV antibody detection, including epitope-blocking ELISA [41]. However, because these assays differ in detection principles, antigen formats, sample panels, and evaluation procedures, their reported detection limits cannot be directly compared with that of the present test strip. The observed analytical sensitivity of the developed strip may be related to the incorporation of selected H- and N-derived epitopes into the recombinant antigen; however, this possibility requires further experimental validation. In the cross-reactivity evaluation, no cross-reactivity was observed with the six heterologous positive serum panels tested, including sera positive for FMD, SP, Bru, Past, MP, and Strep. These findings indicate that the developed strip showed satisfactory analytical performance under the experimental conditions used in this study. When compared with the commercial competitive ELISA, the developed test strip showed an overall agreement of 97.9%, with a Cohen’s kappa coefficient of 0.789. Because the commercial competitive ELISA was used as a comparator assay rather than an independent reference standard, these results represent agreement between the two methods and should not be interpreted as estimates of absolute diagnostic sensitivity or specificity. The high overall agreement observed between the two methods in the serum panel evaluated in the present study supports the feasibility of using the multi-epitope fusion antigen for PPRV antibody detection.

Although the developed test strip showed promising analytical performance, several limitations should be acknowledged. First, one discordant result was observed: the sample was negative by the developed test strip but positive by the commercial ELISA. However, because the original sample-level mapping between the ELISA results and the individual serum identification numbers could not be reliably recovered, the identification number of this discordant sample could not be determined. This discrepancy may be related to differences in analytical sensitivity or to differences in the antibody populations recognized by the two assays. In the absence of virus neutralization test results, the true antibody status of this discordant sample could not be conclusively established. Future studies should therefore include virus neutralization testing for further verification. Second, the individual predicted candidate antigenic regions incorporated into PPRV-H3N3EP were not experimentally validated using epitope-specific assays. Although Western blot analysis confirmed the immunoreactivity of the recombinant fusion protein as a whole, it does not demonstrate that each predicted region independently contributes to antibody recognition. Further studies using synthetic-peptide-based serological assays and cross-lineage sequence conservation analyses are warranted to characterize the antigenicity and relative contribution of each selected region. Third, the performance of the test strip decreased after 5 weeks of storage at room temperature, indicating that its storage stability requires further improvement. Future optimization may include the use of additional stabilizers or freeze-drying strategies. Fourth, the repeatability evaluation was limited to triplicate qualitative testing using strips from a single production batch. Between-batch, inter-day, and inter-operator reproducibility were not evaluated, and quantitative variability in T/C-line intensity was not assessed. Future studies should include multiple independently manufactured batches and quantitative signal analysis to more comprehensively evaluate assay reproducibility. Finally, the number of serum samples evaluated in the present study was relatively limited (*n* = 48), and the commercial ELISA was used as the comparator assay in the absence of virus neutralization test results. Therefore, further validation using larger and more diverse serum panels is required to more comprehensively evaluate the performance and broader applicability of the developed test strip.

The generalizability of these findings should also be evaluated using larger serum panels representing a broader range of breeds and geographic regions. In addition, lineage-defined serum panels representing PPRV lineages I–IV were not included in the present study. Therefore, the cross-lineage detection capability of the PPRV-H3N3EP-based test strip has not yet been experimentally established. Future studies should evaluate the assay using well-characterized sera associated with different PPRV lineages and assess the conservation of the selected H- and N-derived regions among representative strains.

Despite these limitations, the developed test strip is simple to operate, provides results within 15 min, and requires no specialized equipment. These characteristics suggest its potential utility as a preliminary serological screening tool for on-site PPRV antibody detection and post-vaccination antibody monitoring. The present study also supports the feasibility of combining a multi-epitope fusion antigen with a double-antigen sandwich format for the development of rapid antibody-detection assays. Future studies should focus on expanding clinical validation, optimizing antigen conformation, improving room-temperature stability, and conducting more comprehensive assessments of assay reproducibility and cross-lineage applicability.

## 5. Conclusions

In this study, the PPRV-H3N3EP fusion protein with favorable immunoreactivity was constructed and used to develop a colloidal gold immunochromatographic test strip for PPRV antibody detection. The developed strip detected PPRV-positive serum at a dilution of up to 1:64 and showed no cross-reactivity with the six heterologous positive serum panels tested. In the 48 sheep serum samples evaluated, the strip showed an overall agreement of 97.9% with the commercial competitive ELISA, with a Cohen’s kappa coefficient of 0.789. These findings support the potential of the developed strip as a rapid and convenient method for PPRV antibody detection. Further validation using larger and more diverse serum panels is required before its diagnostic performance and broader applicability can be fully established.

## Figures and Tables

**Table 1:** Screening of the Optimal Labeling Conditions for PPRV-H3N3EP Proteins.

No.	Blank 1	Blank 2	1	2	3	4	5	6
Protein (μL)	0	0	5.5	6	6.5	7	7.5	8
Colloidal gold solution (μL)	125	125	125	125	125	125	125	125
10% NaCl (μL)	0	125	125	125	125	125	125	125
Ultrapure water (μL)	125	0	0	0	0	0	0	0

**Table 2:** Within-batch repeatability results of the developed colloidal gold immunochromatographic test strip.

Test Order Per Sample	Serum Sample No.									
	1	2	3	4	5	6	7	8	9	10
1	+	−	+	+	−	−	+	−	−	+
2	+	−	+	+	−	−	+	−	−	+
3	+	−	+	+	−	−	+	−	−	+

+: Positive; −: Negative.

**Table 3:** Agreement between the developed test strip and the commercial competitive ELISA for PPRV antibody detection in 48 sheep serum samples.

Developed Test Strip	ELISA Positive	ELISA Negative	Total
Positive	45	0	45
Negative	1	2	3
Total	46	2	48

## Data Availability

All data generated or analyzed during this study are included in the article/Supplementary Materials.

## Supplementary Materials

The following supporting information can be downloaded at: https://www.mdpi.com/article/10.3390/ani16162545/s1.

## Abbreviations

The following abbreviations are used in this manuscript:

PPR	Peste des Petits Ruminants
PPRV	Peste des Petits Ruminants Virus
WOAH	World Organisation for Animal Health
PCR	Polymerase Chain Reaction
IgG	Immunoglobulin G
NC	Nitrocellulose
bp	Base Pair
IPTG	Isopropyl β-D-1-thiogalactopyranoside
OD	Optical Density
PDI	Polydispersity Index
BCA	Bicinchoninic Acid
BSA	Bovine Serum Albumin
ECL	Enhanced Chemiluminescence
ELISA	Enzyme-Linked Immunosorbent Assay
FMD	Foot-and-Mouth Disease
HRP	Horseradish Peroxidase
LAMP	Loop-Mediated Isothermal Amplification
Ni-NTA	Nickel-Nitrilotriacetic Acid
PBS	Phosphate-Buffered Saline
PEG	Polyethylene Glycol
PVDF	Polyvinylidene Fluoride
SDS-PAGE	Sodium Dodecyl Sulfate–Polyacrylamide Gel Electrophoresis
TBST	Tris-Buffered Saline with Tween
TEM	Transmission Electron Microscopy
