# Supplementary material for: Development of a Colloidal Gold Immunochromatographic Test Strip for PPRV Antibody Detection Based on Antigenic Epitope-Derived Recombinant Protein
Source: Animals (Basel). 2026 Aug 14;16(16):2545. doi: 10.3390/ani16162545 (PMC13508948; doi:10.3390/ani16162545)
Supplement: Supplementary file 1 [file animals-16-02545-s001.zip › Supplementary Figure S1.pdf]

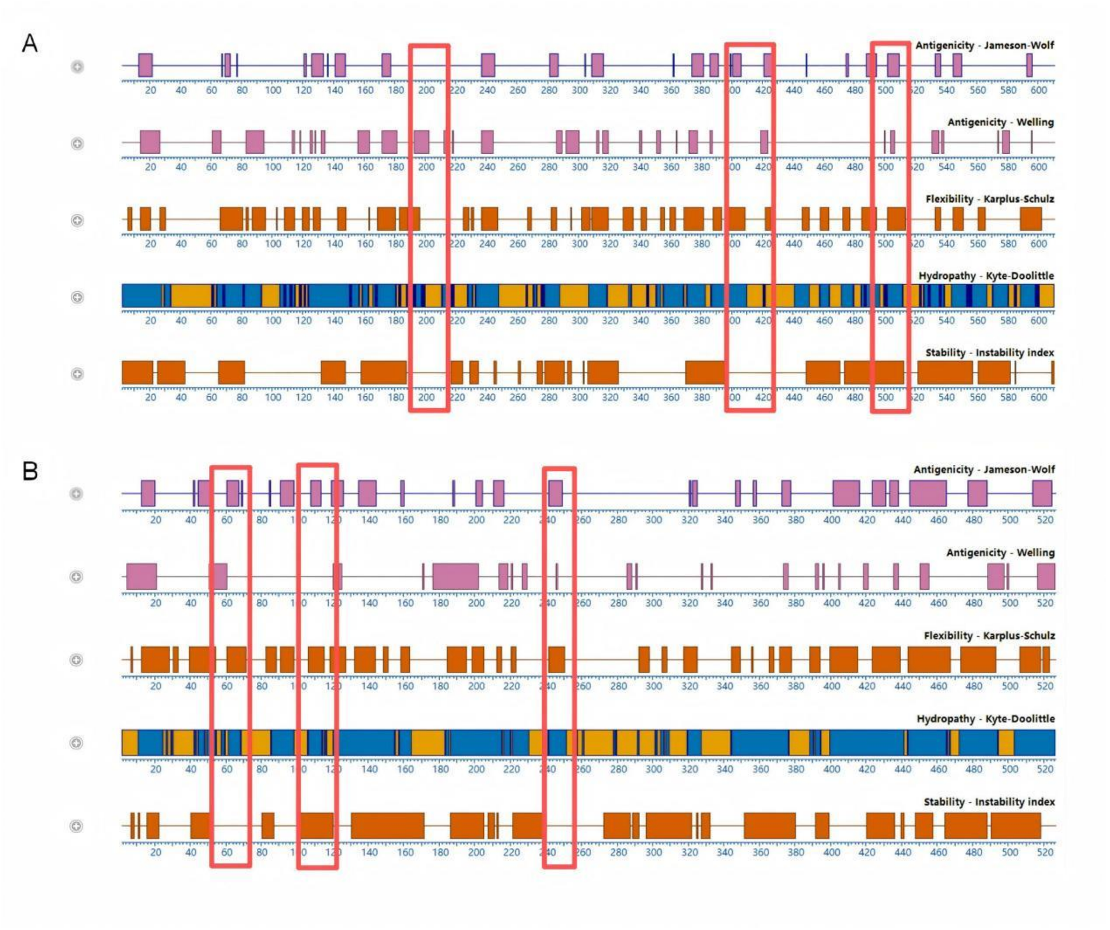

Figure S1. DNASTAR Protean prediction profiles of the PPRV H and N proteins.

(A) Prediction profiles of the PPRV H protein. The red boxes indicate the selected candidate antigenic regions located at residues 195 – 214, 398 – 425, and 496 – 515.

(B) Prediction profiles of the PPRV N protein. The red boxes indicate the selected candidate antigenic regions located at residues 53 – 73, 103 – 122, and 239 – 258.
