# Supplementary material for: Development of a Colloidal Gold Immunochromatographic Test Strip for PPRV Antibody Detection Based on Antigenic Epitope-Derived Recombinant Protein
Source: Animals (Basel). 2026 Aug 14;16(16):2545. doi: 10.3390/ani16162545 (PMC13508948; doi:10.3390/ani16162545)
Supplement: Supplementary file 1 [file animals-16-02545-s001.zip › Supplementary Table S1.pdf]

Supplementary Table S1. Bioinformatically predicted candidate antigenic regions selected from the PPRV H and N proteins for the construction of PPRV-H3N3EP.

**Designation**	**Source protein**	**Protein accession**	**Position (aa)**	**Predicted candidate sequence**
H2	H	AEH25644.1	195–214	KAHFSELTLTLMDLDLEMKH
H16	H	AEH25644.1	398–425	GSGPWSEGRIPAYGVIRVSLDLASDPDV
H1	H	AEH25644.1	496–515	VPIELSRRVDDDIKIGSNMV
N14	N	AEH25639.1	53–73	DRLVRLAGDPDINGSKLTGVM
N1	N	AEH25639.1	103–122	EVVQSTRSQSGLTFASRGAD
N2	N	AEH25639.1	239–258	DIKRTPGNKPRIAEMICDID
