# Supplementary material for: Development of a Colloidal Gold Immunochromatographic Test Strip for PPRV Antibody Detection Based on Antigenic Epitope-Derived Recombinant Protein
Source: Animals (Basel). 2026 Aug 14;16(16):2545. doi: 10.3390/ani16162545 (PMC13508948; doi:10.3390/ani16162545)
Supplement: Supplementary file 1 [file animals-16-02545-s001.zip › Supplementary Table S2.pdf]

**Supplementary Table S2:** Physicochemical characteristics of the PPRV-H3N3EP fusion protein predicted using ExPASy ProtParam.

| Parameter | Predicted value |
|---|---|
| **Number of amino acids** | 209 |
| **Molecular weight** | 21.49 kDa |
| **Theoretical pI** | 6.06 |
| **Molecular formula** | $C_{914}H_{1467}N_{283}O_{301}S_8$ |
| **Total number of atoms** | 2973 |
| **Negatively charged residues (Asp + Glu)** | 23 |
| **Positively charged residues (Arg + Lys)** | 16 |
| **Extinction coefficient, assuming cystines** | 8605 $M^{-1}$ $cm^{-1}$ |
| **Extinction coefficient, assuming reduced Cys** | 8480 $M^{-1}$ $cm^{-1}$ |
| **Estimated half-life in mammalian reticulocytes** | 30 h |
| **Estimated half-life in yeast** | >20 h |
| **Estimated half-life in E. coli** | >10 h |
| **Instability index** | 28.98 |
| **Aliphatic index** | 84.35 |
| **GRAVY** | −0.243 |
